# Trimipraminium maleate

**DOI:** 10.1107/S1600536810000280

**Published:** 2010-01-16

**Authors:** Jerry P. Jasinski, Ray J. Butcher, Q. N. M. Hakim Al-Arique, H. S. Yathirajan, B. Narayana

**Affiliations:** a Department of Chemistry, Keene State College, 229 Main Street, Keene, NH 03435-2001, USA; b Department of Chemistry, Howard University, 525 College Street NW, Washington, DC 20059, USA; c Department of Studies in Chemistry, University of Mysore, Manasagangotri, Mysore 570 006, India; d Department of Studies in Chemistry, Mangalore University, Mangalagangotri 574 199, India

## Abstract

The title compound [systematic name: 3-(10,11-dihydro-5*H*-dibenzo[*b*,*f*]azepin-5-yl)-*N*,*N*,2-trimethyl­propan-1-aminium hydrogen maleate], C_20_H_27_N_2_^+^·C_4_H_3_O_4_^−^, a maleate salt of trimipramine, crystallizes with four independent cation–anion pairs in the asymmetric unit. The trimipramine cation contains a seven-membered azepine ring with two fused benzene rings whose mean planes are separated by 51.7 (1)°. Inter­molecular N—H⋯O and intra­molecular O—H⋯O hydrogen bonds pack the ions into chains along [101]. Additional weak inter­molecular C—H⋯O inter­actions help to influence the twist angles of the mean planes of the benzene rings fused to the azepine ring in the cation. A geometry-optimized MOPAC AM1 theoretical calculation supports these observations.

## Related literature

For refractory depression treatment, see: Broquet, (1999 ▶). For tricyclic anti­depressant treatment, see: Biederman *et al.* (1989 ▶). For treatment of depression, see: Al-Badr, (1983 ▶); Al-Badr & Ibrahim (1979 ▶); Lapierre, (1989 ▶). For protonation of trimipramine salts of maleate, mesylate and hydro­chloride observed by ^1^H, ^13^C and ^15^N NMR, see: Somashekar *et al.* (2004 ▶). For the PMR spectrometric analysis of trimipramine maleate in pharmaceutical preparations, see: Al-Badr & Ibrahim (1979 ▶). For related structures, see: Bindya *et al.* (2007 ▶); Harrison, Bindya *et al.* (2007 ▶); Harrison, Swamy *et al.* (2007 ▶); Jones *et al.* (1978 ▶); Kamel *et al.* (2001 ▶); Portalone *et al.* (2007 ▶); Post *et al.* (1975 ▶); Swamy *et al.* (2007 ▶). For MOPAC AM1 calculations, see: Schmidt & Polik (2007 ▶). For bond-length data, see: Allen *et al.* (1987 ▶).
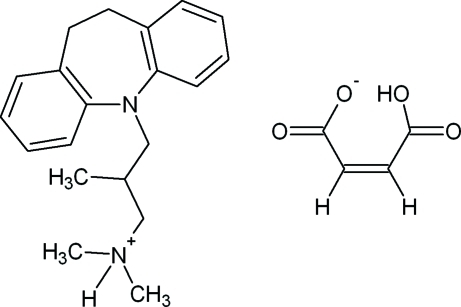


## Experimental

### 

#### Crystal data

C_20_H_27_N_2_^+^·C_4_H_3_O_4_^−^
*M* *_r_* = 410.50
Orthorhombic, 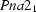

*a* = 19.4356 (4) Å
*b* = 11.0542 (4) Å
*c* = 40.4107 (13) Å
*V* = 8682.0 (5) Å^3^
*Z* = 16
Cu *K*α radiation
μ = 0.69 mm^−1^
*T* = 110 K
0.51 × 0.42 × 0.12 mm

#### Data collection

Oxford Diffraction Xcalibur diffractometer with a Ruby (Gemini Cu) detector
Absorption correction: multi-scan (*CrysAlis RED*; Oxford Diffraction, 2007 ▶) *T*_min_ = 0.576, *T*_max_ = 1.000
27150 measured reflections
13174 (8810) independent reflections
10894 (7507) reflections with *I* > 2σ(*I*)
*R*_int_ = 0.030

#### Refinement

*R*[*F* ^2^ > 2σ(*F* ^2^)] = 0.038
*wR*(*F* ^2^) = 0.095
*S* = 0.96
13174 reflections
1099 parameters
1 restraint
H-atom parameters constrained
Δρ_max_ = 0.25 e Å^−3^
Δρ_min_ = −0.20 e Å^−3^
Absolute structure: Flack (1983 ▶), 8810 Friedel pairs
Flack parameter: −0.14 (13)

### 

Data collection: *CrysAlis PRO* (Oxford Diffraction, 2007 ▶); cell refinement: *CrysAlis PRO*; data reduction: *CrysAlis PRO*; program(s) used to solve structure: *SHELXS97* (Sheldrick, 2008 ▶); program(s) used to refine structure: *SHELXL97* (Sheldrick, 2008 ▶); molecular graphics: *SHELXTL* (Sheldrick, 2008 ▶); software used to prepare material for publication: *SHELXTL*.

## Supplementary Material

Crystal structure: contains datablocks global, I. DOI: 10.1107/S1600536810000280/ng2715sup1.cif

Structure factors: contains datablocks I. DOI: 10.1107/S1600536810000280/ng2715Isup2.hkl

Additional supplementary materials:  crystallographic information; 3D view; checkCIF report

## Figures and Tables

**Table 1:** Hydrogen-bond geometry (Å, °)

*D*—H⋯*A*	*D*—H	H⋯*A*	*D*⋯*A*	*D*—H⋯*A*
O3*A*—H3*A*⋯O1*A*	0.84	1.63	2.437 (2)	161
O3*B*—H3*B*⋯O1*B*	0.84	1.61	2.437 (2)	167
O3*C*—H3*C*⋯O1*C*	0.84	1.58	2.417 (3)	177
O3*D*—H3*D*⋯O1*D*	0.84	1.58	2.422 (3)	178
N2*E*—H2*EB*⋯O2*A*	0.93	1.86	2.736 (3)	156
N2*F*—H2*FB*⋯O2*B*	0.93	1.86	2.737 (3)	156
N2*G*—H2*GB*⋯O2*C*	0.93	1.87	2.760 (3)	158
N2*H*—H2*HB*⋯O4*D*	0.93	1.87	2.751 (3)	158
N2*H*—H2*HB*⋯O3*D*	0.93	2.63	3.312 (3)	131
C17*E*—H17*C*⋯O2*A*	0.98	2.63	3.560 (4)	159
C18*E*—H18*B*⋯O2*D*^i^	0.99	2.28	3.271 (3)	174
C18*F*—H18*C*⋯O4*C*^ii^	0.99	2.31	3.293 (3)	174
C18*G*—H18*E*⋯O2*B*	0.99	2.42	3.404 (3)	171
C18*G*—H18*F*⋯O3*C*^iii^	0.99	2.54	3.453 (3)	154
C18*G*—H18*F*⋯O4*C*^iii^	0.99	2.53	3.432 (3)	152
C18*H*—H18*G*⋯O2*A*	0.99	2.42	3.402 (3)	173
C18*H*—H18*H*⋯O1*D*^iv^	0.99	2.55	3.473 (3)	155
C18*H*—H18*H*⋯O2*D*^iv^	0.99	2.53	3.435 (3)	152
C19*E*—H19*A*⋯O4*D*	0.98	2.62	3.198 (3)	118
C19*F*—H19*E*⋯O4*B*^iv^	0.98	2.54	3.464 (3)	158
C19*G*—H19*I*⋯O4*B*^v^	0.98	2.53	3.476 (3)	163
C19*H*—H19*K*⋯O4*A*^ii^	0.98	2.56	3.516 (3)	164
C20*E*—H20*A*⋯O4*A*^vi^	0.98	2.56	3.488 (3)	159
C20*G*—H20*H*⋯O4*C*^iii^	0.98	2.60	3.479 (3)	149
C20*H*—H20*K*⋯O2*D*^iv^	0.98	2.63	3.497 (3)	148

Symmetry codes: (i) 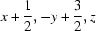; (ii) 
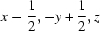
; (iii) 
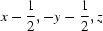
; (iv) 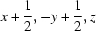; (v) 
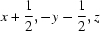
; (vi) 
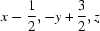
.

**Fig. 1.**
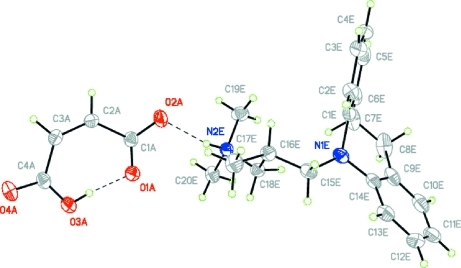
ORTEP drawings of cation E (C20H27N2+) and anion A (C4H3O4-) in the asymmetric unit of (I) showing the atom numbering scheme and 50% probability displacement ellipsoids of non-H atoms. Dashed lines indicate N2E—H2EB···O2A intermolecular and O3A—H3A···O1A intramolecular hydrogen bond interactions.

**Fig. 2.**
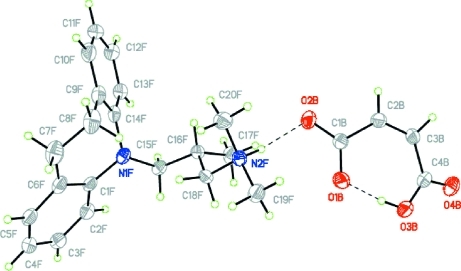
ORTEP drawings of cation F (C20H27N2+) and anion B (C4H3O4-) in the asymmetric unit of (I) showing the atom numbering scheme and 50% probability displacement ellipsoids of non-H atoms. Dashed lines indicate N2F—H2FB···O2B intermolecular and O3B—H3B···O1B intramolecular hydrogen bond interactions.

**Fig. 3.**
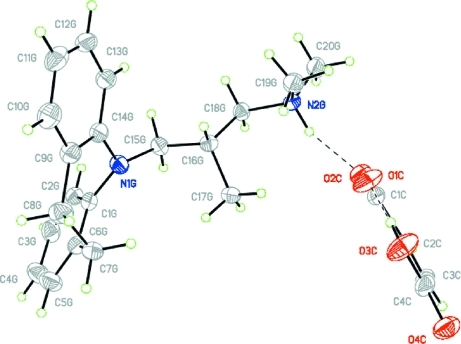
ORTEP drawings of cation G (C20H27N2+) and anion C (C4H3O4-) in the asymmetric unit of (I) showing the atom numbering scheme and 50% probability displacement ellipsoids of non-H atoms. Dashed lines indicate N2G—H2GB···O2C intermolecular and O3C—H3C···O1C intramolecular hydrogen bond interactions.

**Fig. 4.**
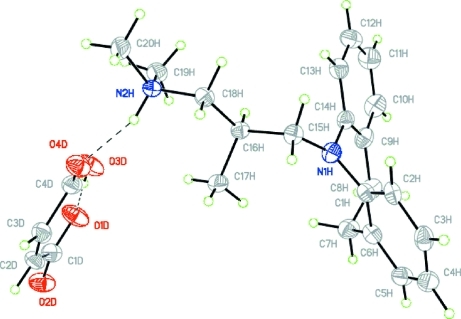
ORTEP drawings of cation H (C20H27N2+) and anion D (C4H3O4-) in the asymmetric unit of (I) showing the atom numbering scheme and 50% probability displacement ellipsoids of non-H atoms. Dashed lines indicate N2H—H2HB···O4C and N2H—H2HB···O3D intermolecular and O3D—H3D···O1D intramolecular hydrogen bond interactions.

**Fig. 5.**
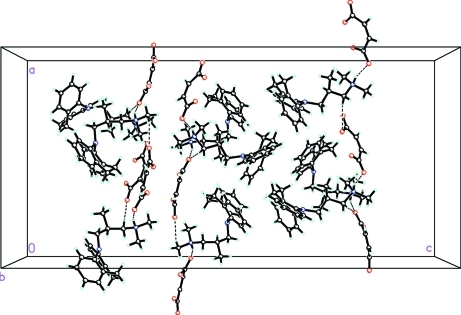
The molecular packing for (I) viewed down the b axis. Dashed lines indicate cation C20H27N2+ and anion C4H3O4- inter and intramolecular hydrogen bond interactions linking the ions into chains along the [101].

## supplementary crystallographic information

### Comment

The title compound is a derivative of trimipramine, chemically, 5-(3-dimethylamino-2-methylpropyl)-10,11-dihydro-5*H*-dibenz(b,f)azepine maleate, a tricyclic antidepressant with sedative and anxiolytic properties and available as stangyl, surmontil, rhotrimine and generic forms. Tricyclic antidepressants are sometimes still used to treat refractory depression that has failed to respond to standard SSRI therapy (Broquet, 1999). Tricyclic antidepressants have been shown to be effective in treating attention-deficit hyperactivity disorder(ADHD) (Biederman *et al.*, 1989). ADHD is thought to be caused by dopamine and norepinephrine shortages in the brain's prefrontal cortex.

Trimipramine is a tricyclic antidepressant of the dibenzazepine class with sedative properties. It also has anticholinergic properties and potentiates the sympathetic response, presumably by blocking the reuptake of norepinephrine which has been released by the presynaptic neurons. It has a quinidine like effect on the heart and an EEG activity similar to that of other tricyclic antidepressants. Trimipramine is a serotonin transport blocker that also blocks norepinephrine uptake. The mechanism of action of trimipramine differs from other tricyclic antidepressants. It is only a moderate reuptake inhibitor of norepinephrine, and a weak reuptake inhibitor of serotonin and dopamine. The spectrum of effects (strong antidepressant activity, sedation and anxiolysis) and side-effects (strong anticholinergic and antiadrenergic side-effects) is the same as with Doxepin. It is also a more effective sedative than amitriptyline. Trimipramine is the only effective drug against insomnia known so far that does not alter the normal sleep architecture.

Trimipramine maleate salt is used in the treatment of depression as well as peptic ulcer and severe chronic pain. A comprehensive description of the title compound is described (Al-Badr, 1983; Lapierre, 1989). The protonation of trimipramine salts of maleate, mesylate and hydrochloride observed by ^1^H, ^13^C and ^15^N NMR spectroscopy is reported (Somashekar *et al.*, 2004). Also, the PMR spectrometric analysis of trimipramine maleate in pharmaceutical preparation is described (Al-Badr & Ibrahim, 1979). The related crystal structures *viz*., imipramine hydrochloride (Post *et al.*, 1975), 5-(3-dimethylammonioprop-1-enylidene)-5*H*-dibenzo[a,d]cycloheptene maleate (Jones *et al.*, 1978), desipraminium picrate (Swamy *et al.*, 2007), desipraminium picrate monohydrate (Harrison, Swamy *et al.*, 2007), imipraminium picrate (Harrison, Bindya *et al.*, 2007), amitriptylinium picrate (Bindya *et al.*, 2007), 5-[3-(dimethylamino)propyl]-10,11-dihydro-5*H*-dibenz[a,d][7]annulen-5-ol (Portalone *et al.*, 2007) have been reported. In view of the importance of the title compound, a crystal structure is reported.

The title compound, C_20_H_27_N_2_^+^. C_4_H_3_O_4_^-^, a maleate salt of trimipramine, crystallizes with four independent cation-anion pairs (E—A, Fig.1; F—B, Fig. 2; G—C, Fig. 3; H—D, Fig. 4) in the asymmetric unit. Bond lengths and bond angles are all within expected ranges (Allen *et al.* 1987). The Trimipramine cation, C_20_H_27_N_2_^+^, contains a seven-membered azepine ring with two fused benzene rings whose mean planes are separated by 51.3 (8)° [E], 51.7 (6)° [F], 49.9 (0)° [G], and 53.9 (0)° [H], respectively. These angles are all less than 57;1(1)° reported for bis((3-(10,11-Dihydro-dibenzo(b,f)azepin-5-yl)-2-methyl-propyl) dimethylammonium) tetrachlorocuprate(ii) (Kamel *et al.* 2001), which contains an identical cation. Intermolecular N—H···O and intramolecular O—H···O hydrogen bonds (Table 1, Fig. 5) pack the ions into chains along the [101]. Additional weak C—H···O intermolecular interactions (Table 1) help to significantly influence the twist angles of the mean planes of the benzene rings fused to the azepine ring in the cation. After a geometry optimimized MOPAC AM1 calculation with WebMO Pro (Schmidt & Polik 2007) on the cation fragment, *in vacuo*, the dihedral angle between the mean planes of the two benzene rings of the azepine group becomes 51.3 (8)°, which is slightly less than the average of the four indepentent cations (51.7 (1)° but nearly 6° less than that observed in the tetrachlorocuprate (ii) analogue compound. These observations support the suggestion that these intra and intermolecular interactions influence crystal packing in the title compound.

### Experimental

The title compound was obtained as a gift sample from *R. L*. Fine Chem, Bangalore, India. The compound was used without further purification. X-ray quality crystals (m.p. 410–412 K) were obtained by slow evaporation from methanol solution.

### Refinement

All of the H atoms were placed in their calculated positions and then refined using the riding model with O–H = 0.84 Å, N–H = 0.93 Å, C–H = 0.95–1.00 Å, and with *U*_iso_(H) = 1.19–2.23*U*_eq_(O), *U*_iso_(H) = 1.18–1.21_eq_(N), *U*_iso_(H) = 1.18–1.51*U*_eq_(C).

### Crystal data

**Table:**

C_20_H_27_N_2_^+^·C_4_H_3_O_4_^−^	*F*(000) = 3520
*M**_r_* = 410.50	*D*_x_ = 1.256 Mg m^−^^3^
Orthorhombic, *P**n**a*2_1_	Cu *K*α radiation, λ = 1.54178 Å
Hall symbol: P 2c -2n	Cell parameters from 11412 reflections
*a* = 19.4356 (4) Å	θ = 4.0–74.2°
*b* = 11.0542 (4) Å	µ = 0.69 mm^−^^1^
*c* = 40.4107 (13) Å	*T* = 110 K
*V* = 8682.0 (5) Å^3^	Plate, colorless
*Z* = 16	0.51 × 0.42 × 0.12 mm

### Data collection

**Table:**

Oxford Diffraction Xcalibur diffractometer with a Ruby (Gemini Cu) detector	13174 (8810) independent reflections
Radiation source: Enhance (Cu) X-ray Source	10894 (7507) reflections with *I* > 2σ(*I*)
graphite	*R*_int_ = 0.030
Detector resolution: 10.5081 pixels mm^-1^	θ_max_ = 74.3°, θ_min_ = 4.2°
ω scans	*h* = −24→16
Absorption correction: multi-scan (*CrysAlis RED*; Oxford Diffraction, 2007)	*k* = −13→10
*T*_min_ = 0.576, *T*_max_ = 1.000	*l* = −49→33
27150 measured reflections	

### Refinement

**Table:**

Refinement on *F*^2^	Secondary atom site location: difference Fourier map
Least-squares matrix: full	Hydrogen site location: inferred from neighbouring sites
*R*[*F*^2^ > 2σ(*F*^2^)] = 0.038	H-atom parameters constrained
*wR*(*F*^2^) = 0.095	*w* = 1/[σ^2^(*F*_o_^2^) + (0.0612*P*)^2^] where *P* = (*F*_o_^2^ + 2*F*_c_^2^)/3
*S* = 0.96	(Δ/σ)_max_ = 0.001
13174 reflections	Δρ_max_ = 0.25 e Å^−^^3^
1099 parameters	Δρ_min_ = −0.20 e Å^−^^3^
1 restraint	Absolute structure: Flack (1983), 8810 Friedel pairs
Primary atom site location: structure-invariant direct methods	Flack parameter: −0.14 (13)

### Special details

**Table:**

Geometry. All e.s.d.'s (except the e.s.d. in the dihedral angle between two l.s. planes) are estimated using the full covariance matrix. The cell e.s.d.'s are taken into account individually in the estimation of e.s.d.'s in distances, angles and torsion angles; correlations between e.s.d.'s in cell parameters are only used when they are defined by crystal symmetry. An approximate (isotropic) treatment of cell e.s.d.'s is used for estimating e.s.d.'s involving l.s. planes.
Refinement. Refinement of *F*^2^ against ALL reflections. The weighted *R*-factor *wR* and goodness of fit *S* are based on *F*^2^, conventional *R*-factors *R* are based on *F*, with *F* set to zero for negative *F*^2^. The threshold expression of *F*^2^ > σ(*F*^2^) is used only for calculating *R*-factors(gt) *etc*. and is not relevant to the choice of reflections for refinement. *R*-factors based on *F*^2^ are statistically about twice as large as those based on *F*, and *R*- factors based on ALL data will be even larger.

### Fractional atomic coordinates and isotropic or equivalent isotropic displacement parameters (Å^2^)

**Table:**

	*x*	*y*	*z*	*U*_iso_*/*U*_eq_	
O1A	0.58175 (9)	0.66955 (15)	0.87821 (5)	0.0343 (4)	
O2A	0.50638 (8)	0.54786 (16)	0.90212 (5)	0.0357 (4)	
O3A	0.70323 (9)	0.66032 (16)	0.86344 (5)	0.0351 (4)	
H3A	0.6600	0.6619	0.8640	0.042*	
O4A	0.78569 (8)	0.52450 (18)	0.86994 (4)	0.0362 (4)	
C1A	0.56195 (11)	0.5666 (2)	0.88763 (6)	0.0271 (5)	
C2A	0.60653 (12)	0.4573 (2)	0.88168 (6)	0.0267 (5)	
H2AA	0.5838	0.3816	0.8837	0.032*	
C3A	0.67296 (12)	0.4509 (2)	0.87401 (6)	0.0268 (5)	
H3AA	0.6904	0.3712	0.8713	0.032*	
C4A	0.72470 (12)	0.5500 (2)	0.86899 (6)	0.0282 (5)	
O1B	0.66282 (9)	0.16974 (15)	0.81349 (5)	0.0348 (4)	
O2B	0.73716 (8)	0.04584 (16)	0.78925 (5)	0.0350 (4)	
O3B	0.54116 (9)	0.16247 (16)	0.82794 (5)	0.0341 (4)	
H3B	0.5811	0.1699	0.8203	0.041*	
O4B	0.45786 (8)	0.02889 (18)	0.82187 (4)	0.0356 (4)	
C1B	0.68199 (11)	0.0647 (2)	0.80384 (6)	0.0254 (5)	
C2B	0.63673 (12)	−0.0427 (2)	0.80977 (6)	0.0281 (5)	
H2BA	0.6588	−0.1190	0.8079	0.034*	
C3B	0.57009 (12)	−0.0470 (2)	0.81730 (6)	0.0279 (5)	
H3BA	0.5518	−0.1263	0.8197	0.033*	
C4B	0.51926 (12)	0.0532 (2)	0.82266 (6)	0.0265 (5)	
O1C	1.00950 (9)	−0.19360 (17)	0.77304 (6)	0.0425 (5)	
O2C	0.93067 (8)	−0.10531 (15)	0.80408 (5)	0.0326 (4)	
O3C	1.12417 (9)	−0.15162 (18)	0.75295 (6)	0.0455 (5)	
H3C	1.0849	−0.1682	0.7603	0.058 (10)*	
O4C	1.20222 (9)	−0.00892 (17)	0.75951 (5)	0.0406 (5)	
C1C	0.98947 (12)	−0.1100 (2)	0.79234 (6)	0.0273 (5)	
C2C	1.03816 (12)	−0.0089 (2)	0.80136 (6)	0.0294 (5)	
H2CA	1.0211	0.0467	0.8173	0.035*	
C3C	1.10112 (12)	0.0141 (2)	0.79044 (6)	0.0310 (5)	
H3CA	1.1215	0.0846	0.7996	0.037*	
C4C	1.14567 (12)	−0.0517 (2)	0.76614 (7)	0.0310 (5)	
O1D	0.12050 (9)	0.34429 (18)	0.93976 (6)	0.0446 (5)	
O2D	0.04202 (9)	0.48725 (17)	0.93376 (6)	0.0442 (5)	
O3D	0.23541 (9)	0.30437 (18)	0.91934 (6)	0.0430 (5)	
H3D	0.1951	0.3177	0.9259	0.096 (15)*	
O4D	0.31337 (8)	0.39596 (16)	0.88852 (5)	0.0351 (4)	
C1D	0.09837 (12)	0.4447 (2)	0.92666 (7)	0.0341 (6)	
C2D	0.14201 (12)	0.5106 (2)	0.90230 (7)	0.0321 (5)	
H2DA	0.1211	0.5808	0.8932	0.039*	
C3D	0.20512 (12)	0.4883 (2)	0.89104 (6)	0.0314 (5)	
H3DA	0.2214	0.5433	0.8748	0.038*	
C4D	0.25439 (12)	0.3894 (2)	0.90020 (6)	0.0297 (5)	
N1E	0.31557 (10)	0.86746 (19)	0.99160 (5)	0.0269 (4)	
N2E	0.41974 (12)	0.74274 (17)	0.90465 (6)	0.0244 (5)	
H2EB	0.4548	0.6876	0.9089	0.029*	
C1E	0.27203 (14)	0.7645 (2)	0.99710 (7)	0.0281 (6)	
C2E	0.28481 (13)	0.6814 (2)	1.02218 (7)	0.0331 (5)	
H2EA	0.3229	0.6922	1.0366	0.040*	
C3E	0.24138 (15)	0.5821 (3)	1.02605 (8)	0.0440 (7)	
H3EA	0.2504	0.5242	1.0428	0.053*	
C4E	0.18535 (15)	0.5679 (3)	1.00553 (9)	0.0506 (8)	
H4EA	0.1554	0.5009	1.0084	0.061*	
C5E	0.17267 (14)	0.6509 (3)	0.98078 (8)	0.0455 (7)	
H5EA	0.1339	0.6401	0.9668	0.055*	
C6E	0.21557 (15)	0.7504 (3)	0.97584 (8)	0.0357 (7)	
C7E	0.20514 (14)	0.8394 (3)	0.94861 (7)	0.0459 (8)	
H7EA	0.1696	0.8079	0.9333	0.055*	
H7EB	0.2486	0.8475	0.9360	0.055*	
C8E	0.18330 (15)	0.9626 (3)	0.96072 (7)	0.0478 (8)	
H8EA	0.1372	0.9540	0.9709	0.057*	
H8EB	0.1779	1.0154	0.9411	0.057*	
C9E	0.22917 (13)	1.0293 (3)	0.98546 (6)	0.0351 (6)	
C10E	0.20910 (14)	1.1469 (3)	0.99337 (8)	0.0430 (7)	
H10A	0.1691	1.1794	0.9831	0.052*	
C11E	0.24513 (15)	1.2180 (3)	1.01551 (10)	0.0504 (8)	
H11A	0.2305	1.2983	1.0201	0.060*	
C12E	0.30230 (15)	1.1711 (3)	1.03075 (9)	0.0474 (7)	
H12A	0.3271	1.2186	1.0463	0.057*	
C13E	0.32394 (13)	1.0545 (3)	1.02359 (7)	0.0366 (6)	
H13A	0.3632	1.0227	1.0346	0.044*	
C14E	0.28892 (12)	0.9827 (2)	1.00042 (6)	0.0291 (5)	
C15E	0.38988 (12)	0.8465 (2)	0.99454 (6)	0.0275 (5)	
H15A	0.4001	0.8121	1.0166	0.033*	
H15B	0.4145	0.9246	0.9926	0.033*	
C16E	0.41544 (15)	0.7607 (2)	0.96804 (7)	0.0281 (6)	
H16A	0.3850	0.6878	0.9675	0.034*	
C17E	0.48914 (13)	0.7210 (3)	0.97591 (8)	0.0363 (6)	
H17A	0.4898	0.6776	0.9970	0.054*	
H17B	0.5188	0.7925	0.9774	0.054*	
H17C	0.5060	0.6677	0.9583	0.054*	
C18E	0.41246 (12)	0.8238 (2)	0.93451 (6)	0.0268 (5)	
H18A	0.3680	0.8672	0.9329	0.032*	
H18B	0.4494	0.8854	0.9337	0.032*	
C19E	0.35584 (13)	0.6736 (2)	0.89740 (7)	0.0358 (6)	
H19A	0.3654	0.6124	0.8804	0.054*	
H19B	0.3202	0.7290	0.8893	0.054*	
H19C	0.3398	0.6336	0.9176	0.054*	
C20E	0.44040 (13)	0.8170 (2)	0.87571 (6)	0.0320 (5)	
H20A	0.4047	0.8774	0.8711	0.048*	
H20B	0.4462	0.7646	0.8563	0.048*	
H20C	0.4839	0.8581	0.8805	0.048*	
N1F	0.93298 (9)	0.36019 (18)	0.70040 (5)	0.0253 (4)	
N2F	0.82441 (11)	0.23990 (17)	0.78662 (6)	0.0240 (5)	
H2FB	0.7893	0.1850	0.7823	0.029*	
C1F	0.96070 (11)	0.4777 (2)	0.69341 (6)	0.0254 (5)	
C2F	0.92522 (13)	0.5557 (2)	0.67222 (6)	0.0316 (5)	
H2FA	0.8855	0.5271	0.6610	0.038*	
C3F	0.94676 (14)	0.6740 (3)	0.66720 (7)	0.0392 (6)	
H3FA	0.9218	0.7257	0.6528	0.047*	
C4F	1.00465 (14)	0.7161 (3)	0.68330 (8)	0.0431 (7)	
H4FA	1.0192	0.7976	0.6807	0.052*	
C5F	1.04099 (13)	0.6379 (2)	0.70325 (7)	0.0365 (6)	
H5FA	1.0812	0.6672	0.7139	0.044*	
C6F	1.02187 (12)	0.5185 (2)	0.70856 (6)	0.0302 (5)	
C7F	1.06982 (14)	0.4437 (3)	0.72995 (7)	0.0405 (6)	
H7FA	1.1128	0.4306	0.7173	0.049*	
H7FB	1.0819	0.4928	0.7496	0.049*	
C8F	1.04523 (13)	0.3215 (3)	0.74211 (6)	0.0395 (6)	
H8FA	1.0805	0.2860	0.7569	0.047*	
H8FB	1.0025	0.3322	0.7551	0.047*	
C9F	1.03169 (14)	0.2364 (3)	0.71393 (7)	0.0314 (6)	
C10F	1.07402 (13)	0.1371 (3)	0.70710 (7)	0.0400 (6)	
H10B	1.1134	0.1232	0.7205	0.048*	
C11F	1.05974 (14)	0.0586 (2)	0.68125 (8)	0.0433 (7)	
H11B	1.0891	−0.0083	0.6770	0.052*	
C12F	1.00240 (14)	0.0786 (3)	0.66168 (8)	0.0394 (6)	
H12B	0.9916	0.0244	0.6442	0.047*	
C13F	0.96095 (13)	0.1777 (2)	0.66772 (6)	0.0312 (5)	
H13B	0.9220	0.1916	0.6540	0.037*	
C14F	0.97509 (15)	0.2575 (2)	0.69345 (7)	0.0257 (6)	
C15F	0.85855 (11)	0.3432 (2)	0.69700 (6)	0.0267 (5)	
H15C	0.8482	0.3103	0.6748	0.032*	
H15D	0.8350	0.4222	0.6992	0.032*	
C16F	0.83121 (14)	0.2554 (2)	0.72370 (7)	0.0248 (6)	
H16B	0.8620	0.1830	0.7248	0.030*	
C17F	0.75860 (12)	0.2147 (3)	0.71426 (7)	0.0339 (6)	
H17D	0.7283	0.2854	0.7129	0.051*	
H17E	0.7411	0.1588	0.7311	0.051*	
H17F	0.7599	0.1737	0.6928	0.051*	
C18F	0.83306 (11)	0.3199 (2)	0.75661 (6)	0.0253 (5)	
H18C	0.7962	0.3817	0.7568	0.030*	
H18D	0.8775	0.3630	0.7585	0.030*	
C19F	0.80369 (13)	0.3156 (2)	0.81538 (6)	0.0318 (5)	
H19D	0.7593	0.3542	0.8107	0.048*	
H19E	0.8386	0.3780	0.8193	0.048*	
H19F	0.7995	0.2645	0.8351	0.048*	
C20F	0.88810 (12)	0.1709 (2)	0.79412 (7)	0.0341 (6)	
H20D	0.9244	0.2270	0.8011	0.051*	
H20E	0.9030	0.1271	0.7743	0.051*	
H20F	0.8789	0.1130	0.8120	0.051*	
N1G	0.75577 (11)	−0.1416 (2)	0.66359 (5)	0.0264 (4)	
N2G	0.84294 (11)	−0.26844 (19)	0.77420 (6)	0.0255 (5)	
H2GB	0.8733	−0.2057	0.7789	0.031*	
C1G	0.73378 (13)	−0.0364 (2)	0.64615 (6)	0.0261 (5)	
C2G	0.66948 (12)	0.0151 (2)	0.65213 (6)	0.0291 (5)	
H2GA	0.6382	−0.0241	0.6667	0.035*	
C3G	0.65072 (13)	0.1227 (3)	0.63715 (6)	0.0353 (6)	
H3GA	0.6076	0.1591	0.6421	0.042*	
C4G	0.69555 (14)	0.1771 (3)	0.61487 (7)	0.0420 (6)	
H4GA	0.6828	0.2500	0.6041	0.050*	
C5G	0.75887 (14)	0.1248 (3)	0.60840 (7)	0.0380 (6)	
H5GA	0.7892	0.1624	0.5931	0.046*	
C6G	0.77886 (12)	0.0179 (2)	0.62392 (6)	0.0290 (5)	
C7G	0.84630 (12)	−0.0437 (2)	0.61698 (6)	0.0304 (5)	
H7GA	0.8702	−0.0601	0.6381	0.037*	
H7GB	0.8758	0.0107	0.6037	0.037*	
C8G	0.83590 (14)	−0.1622 (2)	0.59839 (6)	0.0352 (6)	
H8GA	0.8104	−0.1441	0.5778	0.042*	
H8GB	0.8818	−0.1929	0.5918	0.042*	
C9G	0.79819 (15)	−0.2637 (2)	0.61629 (8)	0.0304 (6)	
C10G	0.80278 (14)	−0.3780 (3)	0.60162 (7)	0.0375 (6)	
H10C	0.8269	−0.3855	0.5813	0.045*	
C11G	0.77363 (14)	−0.4813 (2)	0.61541 (7)	0.0377 (6)	
H11C	0.7772	−0.5573	0.6046	0.045*	
C12G	0.73934 (14)	−0.4708 (3)	0.64528 (7)	0.0343 (6)	
H12C	0.7194	−0.5403	0.6552	0.041*	
C13G	0.73412 (13)	−0.3593 (2)	0.66065 (7)	0.0302 (5)	
H13C	0.7108	−0.3537	0.6812	0.036*	
C14G	0.76249 (14)	−0.2538 (2)	0.64662 (8)	0.0286 (6)	
C15G	0.74368 (12)	−0.1426 (2)	0.69948 (6)	0.0263 (5)	
H15E	0.7049	−0.1976	0.7046	0.032*	
H15F	0.7309	−0.0603	0.7069	0.032*	
C16G	0.80829 (11)	−0.1847 (2)	0.71815 (6)	0.0235 (4)	
H16C	0.8269	−0.2585	0.7070	0.028*	
C17G	0.86398 (12)	−0.0872 (2)	0.71826 (6)	0.0284 (5)	
H17G	0.9069	−0.1213	0.7269	0.043*	
H17H	0.8494	−0.0197	0.7323	0.043*	
H17I	0.8714	−0.0581	0.6956	0.043*	
C18G	0.78581 (11)	−0.2186 (2)	0.75302 (6)	0.0244 (5)	
H18E	0.7665	−0.1460	0.7640	0.029*	
H18F	0.7487	−0.2797	0.7516	0.029*	
C19G	0.88225 (13)	−0.3668 (2)	0.75776 (7)	0.0337 (6)	
H19G	0.9099	−0.3327	0.7397	0.051*	
H19H	0.8502	−0.4267	0.7488	0.051*	
H19I	0.9127	−0.4057	0.7739	0.051*	
C20G	0.81334 (14)	−0.3117 (3)	0.80586 (6)	0.0408 (7)	
H20G	0.8502	−0.3431	0.8200	0.061*	
H20H	0.7800	−0.3761	0.8014	0.061*	
H20I	0.7903	−0.2444	0.8171	0.061*	
N1H	0.49061 (10)	0.37169 (19)	1.02777 (5)	0.0269 (4)	
N2H	0.40102 (11)	0.23369 (18)	0.91833 (5)	0.0243 (4)	
H2HB	0.3701	0.2956	0.9137	0.029*	
C1H	0.51240 (12)	0.4799 (2)	1.04358 (6)	0.0247 (5)	
C2H	0.57518 (11)	0.5346 (2)	1.03544 (6)	0.0263 (5)	
H2HA	0.6063	0.4936	1.0212	0.032*	
C3H	0.59269 (13)	0.6468 (2)	1.04776 (6)	0.0307 (5)	
H3HA	0.6348	0.6838	1.0415	0.037*	
C4H	0.54832 (14)	0.7055 (2)	1.06947 (6)	0.0332 (5)	
H4HA	0.5596	0.7833	1.0779	0.040*	
C5H	0.48754 (13)	0.6495 (2)	1.07870 (6)	0.0329 (5)	
H5HA	0.4581	0.6887	1.0941	0.039*	
C6H	0.46838 (12)	0.5377 (2)	1.06605 (6)	0.0289 (5)	
C7H	0.40374 (13)	0.4722 (2)	1.07605 (7)	0.0338 (6)	
H7HA	0.3769	0.4521	1.0560	0.041*	
H7HB	0.3753	0.5264	1.0900	0.041*	
C8H	0.41876 (14)	0.3567 (2)	1.09509 (7)	0.0356 (6)	
H8HA	0.3744	0.3248	1.1034	0.043*	
H8HB	0.4467	0.3786	1.1147	0.043*	
C9H	0.45548 (15)	0.2541 (2)	1.07722 (8)	0.0304 (6)	
C10H	0.45524 (15)	0.1421 (3)	1.09310 (7)	0.0381 (6)	
H10D	0.4346	0.1365	1.1143	0.046*	
C11H	0.48362 (15)	0.0387 (3)	1.07943 (7)	0.0408 (6)	
H11D	0.4825	−0.0359	1.0911	0.049*	
C12H	0.51347 (14)	0.0462 (3)	1.04862 (8)	0.0360 (6)	
H12D	0.5327	−0.0239	1.0387	0.043*	
C13H	0.51540 (13)	0.1555 (2)	1.03216 (7)	0.0304 (6)	
H13D	0.5362	0.1594	1.0109	0.036*	
C14H	0.48756 (14)	0.2608 (2)	1.04588 (7)	0.0253 (6)	
C15H	0.50098 (12)	0.3667 (2)	0.99175 (6)	0.0259 (5)	
H15G	0.5404	0.3132	0.9867	0.031*	
H15H	0.5120	0.4487	0.9834	0.031*	
C16H	0.43646 (11)	0.3190 (2)	0.97420 (5)	0.0230 (4)	
H16D	0.4199	0.2449	0.9859	0.028*	
C17H	0.37928 (13)	0.4145 (2)	0.97456 (6)	0.0311 (5)	
H17J	0.3368	0.3791	0.9658	0.047*	
H17K	0.3930	0.4835	0.9608	0.047*	
H17L	0.3715	0.4418	0.9973	0.047*	
C18H	0.45769 (11)	0.2851 (2)	0.93916 (6)	0.0249 (5)	
H18G	0.4760	0.3581	0.9280	0.030*	
H18H	0.4954	0.2251	0.9404	0.030*	
C19H	0.36263 (13)	0.1349 (2)	0.93551 (7)	0.0328 (5)	
H19J	0.3954	0.0764	0.9447	0.049*	
H19K	0.3323	0.0940	0.9197	0.049*	
H19L	0.3350	0.1694	0.9535	0.049*	
C20H	0.43019 (14)	0.1898 (3)	0.88633 (6)	0.0410 (7)	
H20J	0.3930	0.1580	0.8724	0.062*	
H20K	0.4638	0.1255	0.8907	0.062*	
H20L	0.4529	0.2570	0.8749	0.062*	

### Atomic displacement parameters (Å^2^)

**Table:**

	*U*^11^	*U*^22^	*U*^33^	*U*^12^	*U*^13^	*U*^23^
O1A	0.0264 (8)	0.0255 (9)	0.0511 (11)	0.0021 (7)	0.0053 (8)	0.0026 (8)
O2A	0.0243 (8)	0.0330 (10)	0.0499 (11)	0.0059 (7)	0.0081 (8)	0.0059 (9)
O3A	0.0276 (8)	0.0313 (10)	0.0465 (10)	−0.0024 (7)	0.0093 (8)	0.0039 (8)
O4A	0.0220 (8)	0.0487 (12)	0.0379 (10)	0.0008 (8)	0.0032 (7)	0.0018 (8)
C1A	0.0234 (11)	0.0286 (12)	0.0292 (12)	0.0029 (9)	−0.0016 (9)	0.0002 (10)
C2A	0.0260 (11)	0.0226 (11)	0.0315 (12)	−0.0015 (9)	−0.0006 (9)	−0.0007 (10)
C3A	0.0287 (11)	0.0250 (12)	0.0269 (11)	0.0041 (9)	0.0009 (9)	0.0000 (10)
C4A	0.0250 (11)	0.0359 (14)	0.0237 (11)	−0.0005 (10)	0.0049 (9)	−0.0016 (10)
O1B	0.0273 (8)	0.0257 (9)	0.0515 (11)	−0.0049 (7)	0.0031 (8)	−0.0010 (8)
O2B	0.0241 (8)	0.0333 (10)	0.0474 (10)	−0.0062 (7)	0.0085 (8)	−0.0049 (8)
O3B	0.0274 (8)	0.0306 (9)	0.0444 (10)	0.0003 (7)	0.0090 (8)	−0.0037 (8)
O4B	0.0223 (8)	0.0465 (11)	0.0380 (9)	−0.0021 (8)	0.0020 (7)	−0.0029 (8)
C1B	0.0199 (10)	0.0263 (12)	0.0299 (11)	−0.0015 (9)	−0.0030 (9)	0.0005 (10)
C2B	0.0254 (11)	0.0239 (12)	0.0349 (12)	0.0003 (9)	0.0003 (10)	−0.0006 (10)
C3B	0.0285 (11)	0.0237 (12)	0.0314 (12)	−0.0056 (9)	0.0043 (10)	−0.0003 (10)
C4B	0.0244 (11)	0.0321 (13)	0.0232 (11)	0.0022 (9)	0.0025 (9)	−0.0017 (10)
O1C	0.0267 (9)	0.0285 (10)	0.0724 (14)	−0.0056 (7)	0.0036 (9)	−0.0166 (10)
O2C	0.0245 (8)	0.0290 (9)	0.0444 (10)	−0.0031 (7)	−0.0001 (7)	−0.0015 (8)
O3C	0.0236 (9)	0.0405 (11)	0.0723 (14)	−0.0030 (8)	0.0073 (9)	−0.0183 (10)
O4C	0.0245 (8)	0.0332 (10)	0.0641 (13)	−0.0037 (7)	−0.0001 (8)	0.0035 (9)
C1C	0.0242 (11)	0.0219 (11)	0.0359 (12)	0.0001 (9)	−0.0075 (9)	0.0030 (10)
C2C	0.0293 (11)	0.0239 (12)	0.0350 (13)	−0.0026 (10)	−0.0073 (10)	−0.0005 (10)
C3C	0.0308 (12)	0.0253 (12)	0.0370 (13)	−0.0035 (10)	−0.0091 (10)	−0.0026 (10)
C4C	0.0208 (11)	0.0259 (12)	0.0462 (15)	0.0011 (9)	−0.0085 (10)	0.0028 (11)
O1D	0.0253 (8)	0.0376 (11)	0.0709 (14)	0.0037 (8)	0.0037 (9)	0.0152 (10)
O2D	0.0228 (8)	0.0337 (10)	0.0760 (14)	0.0026 (7)	−0.0012 (9)	−0.0054 (10)
O3D	0.0252 (9)	0.0327 (11)	0.0710 (14)	0.0042 (8)	0.0023 (9)	0.0125 (10)
O4D	0.0242 (8)	0.0327 (10)	0.0484 (11)	0.0015 (7)	−0.0024 (8)	0.0007 (8)
C1D	0.0219 (11)	0.0301 (13)	0.0502 (15)	−0.0006 (10)	−0.0084 (11)	−0.0060 (12)
C2D	0.0264 (11)	0.0276 (13)	0.0424 (14)	0.0068 (10)	−0.0098 (10)	−0.0009 (11)
C3D	0.0307 (12)	0.0289 (13)	0.0346 (13)	−0.0013 (10)	−0.0061 (10)	0.0006 (11)
C4D	0.0263 (12)	0.0247 (12)	0.0382 (13)	0.0002 (9)	−0.0055 (10)	−0.0046 (11)
N1E	0.0204 (9)	0.0335 (11)	0.0268 (9)	−0.0042 (8)	−0.0018 (8)	0.0008 (8)
N2E	0.0196 (10)	0.0216 (11)	0.0322 (12)	0.0011 (7)	0.0000 (9)	0.0015 (8)
C1E	0.0226 (12)	0.0321 (14)	0.0296 (14)	−0.0005 (9)	0.0041 (11)	−0.0071 (10)
C2E	0.0282 (12)	0.0327 (14)	0.0384 (13)	−0.0020 (10)	0.0061 (10)	−0.0022 (11)
C3E	0.0414 (15)	0.0305 (14)	0.0600 (18)	−0.0016 (12)	0.0175 (14)	−0.0001 (13)
C4E	0.0321 (14)	0.0368 (15)	0.083 (2)	−0.0147 (12)	0.0217 (15)	−0.0220 (16)
C5E	0.0238 (12)	0.0530 (18)	0.0596 (18)	−0.0012 (12)	0.0018 (12)	−0.0288 (15)
C6E	0.0195 (13)	0.0514 (19)	0.0363 (16)	0.0011 (10)	0.0028 (12)	−0.0146 (12)
C7E	0.0246 (12)	0.083 (2)	0.0296 (13)	0.0070 (13)	−0.0057 (10)	−0.0100 (14)
C8E	0.0366 (14)	0.078 (2)	0.0284 (13)	0.0195 (14)	−0.0051 (11)	0.0016 (14)
C9E	0.0290 (12)	0.0480 (16)	0.0282 (12)	−0.0004 (11)	0.0073 (10)	0.0130 (11)
C10E	0.0319 (13)	0.0404 (15)	0.0568 (17)	0.0069 (12)	0.0155 (13)	0.0199 (14)
C11E	0.0326 (14)	0.0306 (14)	0.088 (2)	−0.0045 (12)	0.0231 (15)	−0.0005 (16)
C12E	0.0338 (13)	0.0391 (16)	0.069 (2)	−0.0121 (12)	0.0109 (14)	−0.0151 (14)
C13E	0.0255 (12)	0.0413 (15)	0.0431 (14)	−0.0072 (10)	0.0027 (10)	−0.0027 (12)
C14E	0.0257 (11)	0.0324 (13)	0.0291 (12)	−0.0035 (10)	0.0065 (9)	0.0062 (10)
C15E	0.0220 (11)	0.0317 (13)	0.0287 (11)	−0.0034 (9)	−0.0039 (9)	0.0039 (10)
C16E	0.0228 (13)	0.0286 (14)	0.0329 (15)	0.0006 (9)	−0.0056 (11)	−0.0008 (10)
C17E	0.0258 (13)	0.0383 (14)	0.0447 (16)	0.0067 (11)	−0.0094 (11)	0.0011 (13)
C18E	0.0228 (10)	0.0242 (11)	0.0334 (12)	−0.0002 (9)	−0.0001 (10)	0.0019 (10)
C19E	0.0301 (12)	0.0309 (14)	0.0462 (15)	−0.0039 (11)	−0.0099 (11)	−0.0027 (12)
C20E	0.0286 (12)	0.0362 (14)	0.0313 (12)	0.0050 (10)	0.0002 (10)	0.0054 (11)
N1F	0.0175 (8)	0.0324 (11)	0.0261 (9)	0.0002 (8)	−0.0006 (7)	−0.0015 (8)
N2F	0.0185 (10)	0.0221 (11)	0.0314 (12)	−0.0036 (7)	−0.0014 (9)	−0.0020 (8)
C1F	0.0214 (10)	0.0295 (12)	0.0253 (11)	0.0016 (9)	0.0057 (9)	−0.0057 (9)
C2F	0.0257 (11)	0.0364 (14)	0.0329 (12)	0.0013 (10)	0.0035 (10)	0.0006 (11)
C3F	0.0336 (13)	0.0338 (14)	0.0503 (15)	0.0059 (11)	0.0109 (12)	0.0055 (12)
C4F	0.0322 (14)	0.0311 (14)	0.0660 (19)	−0.0030 (12)	0.0179 (13)	−0.0052 (14)
C5F	0.0234 (11)	0.0376 (14)	0.0486 (15)	−0.0035 (10)	0.0107 (11)	−0.0170 (12)
C6F	0.0226 (10)	0.0391 (14)	0.0289 (11)	−0.0005 (10)	0.0057 (9)	−0.0074 (11)
C7F	0.0297 (12)	0.0585 (18)	0.0333 (13)	−0.0083 (12)	−0.0058 (10)	−0.0035 (12)
C8F	0.0274 (12)	0.0620 (18)	0.0292 (12)	−0.0051 (12)	−0.0050 (10)	0.0106 (12)
C9F	0.0223 (13)	0.0397 (15)	0.0323 (15)	−0.0026 (10)	0.0002 (11)	0.0115 (11)
C10F	0.0224 (11)	0.0425 (15)	0.0549 (16)	−0.0018 (11)	−0.0008 (11)	0.0195 (13)
C11F	0.0302 (13)	0.0257 (13)	0.074 (2)	0.0031 (10)	0.0089 (13)	0.0070 (13)
C12F	0.0316 (13)	0.0329 (14)	0.0539 (17)	−0.0013 (11)	0.0083 (12)	−0.0014 (13)
C13F	0.0263 (11)	0.0306 (13)	0.0368 (13)	−0.0019 (10)	0.0015 (10)	0.0011 (11)
C14F	0.0214 (12)	0.0316 (14)	0.0241 (13)	−0.0044 (9)	0.0027 (10)	0.0059 (9)
C15F	0.0205 (10)	0.0303 (12)	0.0293 (11)	0.0019 (9)	−0.0006 (9)	−0.0008 (10)
C16F	0.0205 (12)	0.0222 (13)	0.0317 (15)	−0.0012 (8)	0.0004 (11)	0.0001 (9)
C17F	0.0249 (12)	0.0359 (14)	0.0408 (15)	−0.0072 (11)	−0.0031 (10)	−0.0003 (13)
C18F	0.0228 (10)	0.0184 (11)	0.0347 (12)	−0.0014 (8)	0.0013 (9)	0.0016 (10)
C19F	0.0285 (12)	0.0348 (14)	0.0322 (12)	−0.0042 (10)	0.0030 (10)	−0.0069 (11)
C20F	0.0274 (11)	0.0316 (14)	0.0433 (14)	0.0022 (10)	−0.0058 (11)	0.0011 (11)
N1G	0.0293 (10)	0.0263 (11)	0.0237 (10)	0.0004 (8)	0.0006 (8)	0.0018 (8)
N2G	0.0233 (10)	0.0254 (10)	0.0279 (11)	−0.0062 (8)	−0.0036 (9)	0.0036 (9)
C1G	0.0293 (12)	0.0257 (13)	0.0234 (11)	−0.0037 (10)	−0.0045 (10)	−0.0001 (10)
C2G	0.0257 (11)	0.0343 (13)	0.0272 (11)	−0.0063 (10)	−0.0020 (9)	0.0016 (10)
C3G	0.0286 (11)	0.0430 (15)	0.0345 (13)	0.0049 (11)	−0.0045 (10)	0.0035 (11)
C4G	0.0444 (14)	0.0381 (15)	0.0435 (14)	0.0039 (12)	−0.0038 (12)	0.0150 (12)
C5G	0.0371 (13)	0.0410 (15)	0.0358 (13)	−0.0041 (11)	0.0052 (11)	0.0111 (11)
C6G	0.0293 (11)	0.0305 (13)	0.0272 (11)	−0.0065 (10)	−0.0011 (9)	−0.0013 (10)
C7G	0.0291 (11)	0.0332 (13)	0.0289 (12)	−0.0067 (10)	0.0031 (10)	0.0050 (10)
C8G	0.0386 (13)	0.0355 (14)	0.0315 (12)	−0.0062 (11)	0.0088 (11)	−0.0019 (11)
C9G	0.0287 (13)	0.0304 (14)	0.0321 (14)	−0.0014 (10)	−0.0019 (12)	0.0010 (10)
C10G	0.0365 (13)	0.0394 (15)	0.0365 (13)	0.0008 (11)	−0.0003 (11)	−0.0068 (12)
C11G	0.0368 (13)	0.0311 (14)	0.0451 (15)	0.0014 (11)	−0.0083 (12)	−0.0098 (12)
C12G	0.0304 (13)	0.0283 (14)	0.0441 (15)	−0.0061 (10)	−0.0101 (12)	0.0066 (12)
C13G	0.0241 (11)	0.0326 (13)	0.0339 (13)	−0.0035 (10)	−0.0020 (10)	0.0046 (11)
C14G	0.0232 (14)	0.0323 (15)	0.0303 (15)	−0.0031 (9)	−0.0047 (11)	0.0040 (10)
C15G	0.0263 (11)	0.0278 (12)	0.0249 (11)	0.0016 (9)	−0.0002 (9)	0.0028 (9)
C16G	0.0222 (10)	0.0221 (11)	0.0262 (11)	−0.0009 (9)	0.0015 (9)	0.0001 (9)
C17G	0.0285 (11)	0.0251 (12)	0.0314 (12)	−0.0041 (9)	−0.0008 (10)	0.0060 (10)
C18G	0.0197 (10)	0.0285 (12)	0.0251 (11)	0.0007 (10)	−0.0007 (9)	0.0014 (10)
C19G	0.0357 (13)	0.0215 (12)	0.0439 (14)	0.0018 (10)	−0.0109 (11)	0.0007 (11)
C20G	0.0375 (14)	0.0577 (19)	0.0271 (12)	−0.0180 (13)	−0.0049 (11)	0.0136 (13)
N1H	0.0280 (10)	0.0287 (11)	0.0240 (10)	−0.0024 (8)	0.0014 (8)	0.0000 (8)
N2H	0.0245 (10)	0.0238 (10)	0.0245 (11)	0.0056 (8)	−0.0010 (8)	−0.0026 (8)
C1H	0.0251 (12)	0.0262 (13)	0.0230 (11)	0.0027 (9)	−0.0028 (9)	0.0044 (10)
C2H	0.0221 (10)	0.0341 (13)	0.0226 (10)	0.0008 (9)	−0.0041 (9)	0.0041 (9)
C3H	0.0321 (11)	0.0316 (13)	0.0283 (11)	−0.0064 (10)	−0.0078 (9)	0.0083 (10)
C4H	0.0434 (13)	0.0216 (11)	0.0346 (12)	−0.0019 (10)	−0.0086 (11)	0.0052 (10)
C5H	0.0370 (13)	0.0299 (13)	0.0318 (12)	0.0081 (10)	−0.0019 (10)	−0.0027 (10)
C6H	0.0259 (11)	0.0312 (13)	0.0295 (12)	0.0037 (10)	−0.0022 (9)	0.0033 (10)
C7H	0.0289 (12)	0.0372 (14)	0.0354 (13)	0.0024 (10)	0.0061 (10)	−0.0061 (11)
C8H	0.0382 (13)	0.0358 (14)	0.0329 (13)	−0.0092 (11)	0.0097 (11)	−0.0022 (11)
C9H	0.0294 (13)	0.0331 (15)	0.0286 (14)	−0.0081 (10)	0.0017 (11)	−0.0025 (10)
C10H	0.0427 (14)	0.0404 (15)	0.0313 (13)	−0.0078 (12)	0.0050 (12)	0.0025 (11)
C11H	0.0484 (16)	0.0316 (14)	0.0424 (15)	−0.0027 (12)	−0.0026 (12)	0.0095 (12)
C12H	0.0329 (14)	0.0315 (14)	0.0435 (16)	0.0053 (11)	0.0016 (12)	−0.0005 (12)
C13H	0.0287 (12)	0.0329 (13)	0.0296 (12)	−0.0006 (10)	−0.0005 (10)	0.0003 (11)
C14H	0.0234 (13)	0.0251 (13)	0.0274 (14)	−0.0032 (9)	−0.0017 (11)	−0.0019 (9)
C15H	0.0254 (10)	0.0258 (12)	0.0265 (11)	−0.0034 (9)	0.0009 (9)	0.0011 (10)
C16H	0.0230 (10)	0.0211 (11)	0.0249 (11)	−0.0005 (8)	0.0012 (9)	−0.0008 (9)
C17H	0.0287 (12)	0.0298 (13)	0.0349 (13)	0.0062 (10)	−0.0017 (10)	−0.0074 (11)
C18H	0.0164 (10)	0.0292 (12)	0.0292 (12)	0.0007 (9)	0.0018 (9)	−0.0011 (11)
C19H	0.0365 (12)	0.0188 (11)	0.0432 (14)	−0.0034 (10)	−0.0092 (11)	0.0002 (11)
C20H	0.0351 (13)	0.061 (2)	0.0275 (12)	0.0180 (13)	−0.0043 (11)	−0.0120 (13)

### Geometric parameters (Å, °)

**Table:**

O1A—C1A	1.260 (3)	C12F—H12B	0.9500
O2A—C1A	1.246 (3)	C13F—C14F	1.391 (4)
O3A—C4A	1.308 (3)	C13F—H13B	0.9500
O3A—H3A	0.8400	C15F—C16F	1.545 (4)
O4A—C4A	1.219 (3)	C15F—H15C	0.9900
C1A—C2A	1.506 (3)	C15F—H15D	0.9900
C2A—C3A	1.330 (3)	C16F—C18F	1.509 (4)
C2A—H2AA	0.9500	C16F—C17F	1.530 (3)
C3A—C4A	1.501 (3)	C16F—H16B	1.0000
C3A—H3AA	0.9500	C17F—H17D	0.9800
O1B—C1B	1.280 (3)	C17F—H17E	0.9800
O2B—C1B	1.241 (3)	C17F—H17F	0.9800
O3B—C4B	1.299 (3)	C18F—H18C	0.9900
O3B—H3B	0.8400	C18F—H18D	0.9900
O4B—C4B	1.224 (3)	C19F—H19D	0.9800
C1B—C2B	1.497 (3)	C19F—H19E	0.9800
C2B—C3B	1.331 (3)	C19F—H19F	0.9800
C2B—H2BA	0.9500	C20F—H20D	0.9800
C3B—C4B	1.500 (3)	C20F—H20E	0.9800
C3B—H3BA	0.9500	C20F—H20F	0.9800
O1C—C1C	1.270 (3)	N1G—C14G	1.423 (3)
O2C—C1C	1.238 (3)	N1G—C1G	1.426 (3)
O3C—C4C	1.296 (3)	N1G—C15G	1.469 (3)
O3C—H3C	0.8400	N2G—C20G	1.482 (3)
O4C—C4C	1.226 (3)	N2G—C19G	1.485 (3)
C1C—C2C	1.509 (3)	N2G—C18G	1.506 (3)
C2C—C3C	1.325 (3)	N2G—H2GB	0.9300
C2C—H2CA	0.9500	C1G—C6G	1.391 (4)
C3C—C4C	1.498 (4)	C1G—C2G	1.394 (4)
C3C—H3CA	0.9500	C2G—C3G	1.384 (4)
O1D—C1D	1.302 (3)	C2G—H2GA	0.9500
O2D—C1D	1.226 (3)	C3G—C4G	1.390 (4)
O3D—C4D	1.272 (3)	C3G—H3GA	0.9500
O3D—H3D	0.8400	C4G—C5G	1.385 (4)
O4D—C4D	1.242 (3)	C4G—H4GA	0.9500
C1D—C2D	1.490 (4)	C5G—C6G	1.393 (4)
C2D—C3D	1.331 (3)	C5G—H5GA	0.9500
C2D—H2DA	0.9500	C6G—C7G	1.503 (3)
C3D—C4D	1.499 (3)	C7G—C8G	1.524 (4)
C3D—H3DA	0.9500	C7G—H7GA	0.9900
N1E—C14E	1.421 (3)	C7G—H7GB	0.9900
N1E—C1E	1.436 (3)	C8G—C9G	1.523 (4)
N1E—C15E	1.468 (3)	C8G—H8GA	0.9900
N2E—C20E	1.484 (3)	C8G—H8GB	0.9900
N2E—C19E	1.488 (3)	C9G—C10G	1.399 (4)
N2E—C18E	1.510 (3)	C9G—C14G	1.413 (4)
N2E—H2EB	0.9300	C10G—C11G	1.391 (4)
C1E—C2E	1.390 (4)	C10G—H10C	0.9500
C1E—C6E	1.402 (4)	C11G—C12G	1.383 (4)
C2E—C3E	1.393 (4)	C11G—H11C	0.9500
C2E—H2EA	0.9500	C12G—C13G	1.384 (4)
C3E—C4E	1.377 (5)	C12G—H12C	0.9500
C3E—H3EA	0.9500	C13G—C14G	1.409 (4)
C4E—C5E	1.380 (5)	C13G—H13C	0.9500
C4E—H4EA	0.9500	C15G—C16G	1.537 (3)
C5E—C6E	1.394 (4)	C15G—H15E	0.9900
C5E—H5EA	0.9500	C15G—H15F	0.9900
C6E—C7E	1.490 (5)	C16G—C18G	1.522 (3)
C7E—C8E	1.509 (5)	C16G—C17G	1.527 (3)
C7E—H7EA	0.9900	C16G—H16C	1.0000
C7E—H7EB	0.9900	C17G—H17G	0.9800
C8E—C9E	1.529 (4)	C17G—H17H	0.9800
C8E—H8EA	0.9900	C17G—H17I	0.9800
C8E—H8EB	0.9900	C18G—H18E	0.9900
C9E—C10E	1.395 (4)	C18G—H18F	0.9900
C9E—C14E	1.406 (4)	C19G—H19G	0.9800
C10E—C11E	1.382 (5)	C19G—H19H	0.9800
C10E—H10A	0.9500	C19G—H19I	0.9800
C11E—C12E	1.372 (5)	C20G—H20G	0.9800
C11E—H11A	0.9500	C20G—H20H	0.9800
C12E—C13E	1.386 (4)	C20G—H20I	0.9800
C12E—H12A	0.9500	N1H—C1H	1.421 (3)
C13E—C14E	1.404 (4)	N1H—C14H	1.429 (3)
C13E—H13A	0.9500	N1H—C15H	1.470 (3)
C15E—C16E	1.514 (4)	N2H—C20H	1.493 (3)
C15E—H15A	0.9900	N2H—C19H	1.494 (3)
C15E—H15B	0.9900	N2H—C18H	1.498 (3)
C16E—C18E	1.525 (4)	N2H—H2HB	0.9300
C16E—C17E	1.531 (4)	C1H—C2H	1.401 (3)
C16E—H16A	1.0000	C1H—C6H	1.402 (4)
C17E—H17A	0.9800	C2H—C3H	1.379 (4)
C17E—H17B	0.9800	C2H—H2HA	0.9500
C17E—H17C	0.9800	C3H—C4H	1.391 (4)
C18E—H18A	0.9900	C3H—H3HA	0.9500
C18E—H18B	0.9900	C4H—C5H	1.385 (4)
C19E—H19A	0.9800	C4H—H4HA	0.9500
C19E—H19B	0.9800	C5H—C6H	1.388 (4)
C19E—H19C	0.9800	C5H—H5HA	0.9500
C20E—H20A	0.9800	C6H—C7H	1.505 (3)
C20E—H20B	0.9800	C7H—C8H	1.520 (4)
C20E—H20C	0.9800	C7H—H7HA	0.9900
N1F—C14F	1.427 (3)	C7H—H7HB	0.9900
N1F—C1F	1.435 (3)	C8H—C9H	1.522 (4)
N1F—C15F	1.465 (3)	C8H—H8HA	0.9900
N2F—C20F	1.485 (3)	C8H—H8HB	0.9900
N2F—C19F	1.488 (3)	C9H—C10H	1.395 (4)
N2F—C18F	1.511 (3)	C9H—C14H	1.413 (4)
N2F—H2FB	0.9300	C10H—C11H	1.384 (4)
C1F—C2F	1.397 (4)	C10H—H10D	0.9500
C1F—C6F	1.411 (3)	C11H—C12H	1.376 (4)
C2F—C3F	1.388 (4)	C11H—H11D	0.9500
C2F—H2FA	0.9500	C12H—C13H	1.380 (4)
C3F—C4F	1.381 (4)	C12H—H12D	0.9500
C3F—H3FA	0.9500	C13H—C14H	1.398 (4)
C4F—C5F	1.377 (4)	C13H—H13D	0.9500
C4F—H4FA	0.9500	C15H—C16H	1.534 (3)
C5F—C6F	1.388 (4)	C15H—H15G	0.9900
C5F—H5FA	0.9500	C15H—H15H	0.9900
C6F—C7F	1.517 (4)	C16H—C18H	1.521 (3)
C7F—C8F	1.515 (4)	C16H—C17H	1.532 (3)
C7F—H7FA	0.9900	C16H—H16D	1.0000
C7F—H7FB	0.9900	C17H—H17J	0.9800
C8F—C9F	1.500 (4)	C17H—H17K	0.9800
C8F—H8FA	0.9900	C17H—H17L	0.9800
C8F—H8FB	0.9900	C18H—H18G	0.9900
C9F—C14F	1.396 (4)	C18H—H18H	0.9900
C9F—C10F	1.399 (4)	C19H—H19J	0.9800
C10F—C11F	1.386 (4)	C19H—H19K	0.9800
C10F—H10B	0.9500	C19H—H19L	0.9800
C11F—C12F	1.384 (4)	C20H—H20J	0.9800
C11F—H11B	0.9500	C20H—H20K	0.9800
C12F—C13F	1.382 (4)	C20H—H20L	0.9800
			
C4A—O3A—H3A	109.5	C17F—C16F—H16B	109.1
O2A—C1A—O1A	123.8 (2)	C15F—C16F—H16B	109.1
O2A—C1A—C2A	116.1 (2)	C16F—C17F—H17D	109.5
O1A—C1A—C2A	120.0 (2)	C16F—C17F—H17E	109.5
C3A—C2A—C1A	129.7 (2)	H17D—C17F—H17E	109.5
C3A—C2A—H2AA	115.2	C16F—C17F—H17F	109.5
C1A—C2A—H2AA	115.2	H17D—C17F—H17F	109.5
C2A—C3A—C4A	130.0 (2)	H17E—C17F—H17F	109.5
C2A—C3A—H3AA	115.0	C16F—C18F—N2F	115.35 (19)
C4A—C3A—H3AA	115.0	C16F—C18F—H18C	108.4
O4A—C4A—O3A	122.1 (2)	N2F—C18F—H18C	108.4
O4A—C4A—C3A	118.6 (2)	C16F—C18F—H18D	108.4
O3A—C4A—C3A	119.3 (2)	N2F—C18F—H18D	108.4
C4B—O3B—H3B	109.5	H18C—C18F—H18D	107.5
O2B—C1B—O1B	123.3 (2)	N2F—C19F—H19D	109.5
O2B—C1B—C2B	116.8 (2)	N2F—C19F—H19E	109.5
O1B—C1B—C2B	120.0 (2)	H19D—C19F—H19E	109.5
C3B—C2B—C1B	129.6 (2)	N2F—C19F—H19F	109.5
C3B—C2B—H2BA	115.2	H19D—C19F—H19F	109.5
C1B—C2B—H2BA	115.2	H19E—C19F—H19F	109.5
C2B—C3B—C4B	130.3 (2)	N2F—C20F—H20D	109.5
C2B—C3B—H3BA	114.8	N2F—C20F—H20E	109.5
C4B—C3B—H3BA	114.8	H20D—C20F—H20E	109.5
O4B—C4B—O3B	121.9 (2)	N2F—C20F—H20F	109.5
O4B—C4B—C3B	118.5 (2)	H20D—C20F—H20F	109.5
O3B—C4B—C3B	119.7 (2)	H20E—C20F—H20F	109.5
C4C—O3C—H3C	109.5	C14G—N1G—C1G	120.0 (2)
O2C—C1C—O1C	123.3 (2)	C14G—N1G—C15G	118.9 (2)
O2C—C1C—C2C	117.1 (2)	C1G—N1G—C15G	116.5 (2)
O1C—C1C—C2C	119.7 (2)	C20G—N2G—C19G	110.5 (2)
C3C—C2C—C1C	129.9 (2)	C20G—N2G—C18G	108.8 (2)
C3C—C2C—H2CA	115.1	C19G—N2G—C18G	113.1 (2)
C1C—C2C—H2CA	115.1	C20G—N2G—H2GB	108.1
C2C—C3C—C4C	131.2 (2)	C19G—N2G—H2GB	108.1
C2C—C3C—H3CA	114.4	C18G—N2G—H2GB	108.1
C4C—C3C—H3CA	114.4	C6G—C1G—C2G	120.0 (2)
O4C—C4C—O3C	121.9 (2)	C6G—C1G—N1G	118.8 (2)
O4C—C4C—C3C	118.3 (2)	C2G—C1G—N1G	121.1 (2)
O3C—C4C—C3C	119.8 (2)	C3G—C2G—C1G	120.8 (2)
C4D—O3D—H3D	109.5	C3G—C2G—H2GA	119.6
O2D—C1D—O1D	121.8 (3)	C1G—C2G—H2GA	119.6
O2D—C1D—C2D	118.4 (2)	C2G—C3G—C4G	119.3 (2)
O1D—C1D—C2D	119.8 (2)	C2G—C3G—H3GA	120.3
C3D—C2D—C1D	131.3 (2)	C4G—C3G—H3GA	120.3
C3D—C2D—H2DA	114.4	C5G—C4G—C3G	120.0 (3)
C1D—C2D—H2DA	114.4	C5G—C4G—H4GA	120.0
C2D—C3D—C4D	129.7 (2)	C3G—C4G—H4GA	120.0
C2D—C3D—H3DA	115.1	C4G—C5G—C6G	121.1 (2)
C4D—C3D—H3DA	115.1	C4G—C5G—H5GA	119.4
O4D—C4D—O3D	122.8 (2)	C6G—C5G—H5GA	119.4
O4D—C4D—C3D	117.0 (2)	C1G—C6G—C5G	118.8 (2)
O3D—C4D—C3D	120.2 (2)	C1G—C6G—C7G	118.3 (2)
C14E—N1E—C1E	117.2 (2)	C5G—C6G—C7G	122.9 (2)
C14E—N1E—C15E	118.7 (2)	C6G—C7G—C8G	111.4 (2)
C1E—N1E—C15E	116.2 (2)	C6G—C7G—H7GA	109.3
C20E—N2E—C19E	110.8 (2)	C8G—C7G—H7GA	109.3
C20E—N2E—C18E	109.06 (18)	C6G—C7G—H7GB	109.3
C19E—N2E—C18E	112.6 (2)	C8G—C7G—H7GB	109.3
C20E—N2E—H2EB	108.1	H7GA—C7G—H7GB	108.0
C19E—N2E—H2EB	108.1	C9G—C8G—C7G	117.6 (2)
C18E—N2E—H2EB	108.1	C9G—C8G—H8GA	107.9
C2E—C1E—C6E	120.9 (3)	C7G—C8G—H8GA	107.9
C2E—C1E—N1E	122.1 (2)	C9G—C8G—H8GB	107.9
C6E—C1E—N1E	117.0 (3)	C7G—C8G—H8GB	107.9
C1E—C2E—C3E	119.6 (3)	H8GA—C8G—H8GB	107.2
C1E—C2E—H2EA	120.2	C10G—C9G—C14G	118.0 (3)
C3E—C2E—H2EA	120.2	C10G—C9G—C8G	115.7 (3)
C4E—C3E—C2E	120.1 (3)	C14G—C9G—C8G	126.3 (2)
C4E—C3E—H3EA	120.0	C11G—C10G—C9G	123.1 (3)
C2E—C3E—H3EA	120.0	C11G—C10G—H10C	118.5
C3E—C4E—C5E	120.1 (3)	C9G—C10G—H10C	118.5
C3E—C4E—H4EA	119.9	C12G—C11G—C10G	118.5 (3)
C5E—C4E—H4EA	119.9	C12G—C11G—H11C	120.7
C4E—C5E—C6E	121.4 (3)	C10G—C11G—H11C	120.7
C4E—C5E—H5EA	119.3	C11G—C12G—C13G	120.1 (3)
C6E—C5E—H5EA	119.3	C11G—C12G—H12C	120.0
C5E—C6E—C1E	117.9 (3)	C13G—C12G—H12C	120.0
C5E—C6E—C7E	123.0 (3)	C12G—C13G—C14G	121.9 (3)
C1E—C6E—C7E	119.1 (3)	C12G—C13G—H13C	119.1
C6E—C7E—C8E	113.3 (2)	C14G—C13G—H13C	119.1
C6E—C7E—H7EA	108.9	C13G—C14G—C9G	118.5 (2)
C8E—C7E—H7EA	108.9	C13G—C14G—N1G	119.4 (3)
C6E—C7E—H7EB	108.9	C9G—C14G—N1G	122.0 (2)
C8E—C7E—H7EB	108.9	N1G—C15G—C16G	110.87 (19)
H7EA—C7E—H7EB	107.7	N1G—C15G—H15E	109.5
C7E—C8E—C9E	118.9 (2)	C16G—C15G—H15E	109.5
C7E—C8E—H8EA	107.6	N1G—C15G—H15F	109.5
C9E—C8E—H8EA	107.6	C16G—C15G—H15F	109.5
C7E—C8E—H8EB	107.6	H15E—C15G—H15F	108.1
C9E—C8E—H8EB	107.6	C18G—C16G—C17G	112.0 (2)
H8EA—C8E—H8EB	107.0	C18G—C16G—C15G	107.13 (18)
C10E—C9E—C14E	118.3 (3)	C17G—C16G—C15G	111.55 (19)
C10E—C9E—C8E	115.8 (3)	C18G—C16G—H16C	108.7
C14E—C9E—C8E	125.9 (3)	C17G—C16G—H16C	108.7
C11E—C10E—C9E	122.5 (3)	C15G—C16G—H16C	108.7
C11E—C10E—H10A	118.8	C16G—C17G—H17G	109.5
C9E—C10E—H10A	118.8	C16G—C17G—H17H	109.5
C12E—C11E—C10E	119.1 (3)	H17G—C17G—H17H	109.5
C12E—C11E—H11A	120.5	C16G—C17G—H17I	109.5
C10E—C11E—H11A	120.5	H17G—C17G—H17I	109.5
C11E—C12E—C13E	120.2 (3)	H17H—C17G—H17I	109.5
C11E—C12E—H12A	119.9	N2G—C18G—C16G	113.87 (19)
C13E—C12E—H12A	119.9	N2G—C18G—H18E	108.8
C12E—C13E—C14E	121.2 (3)	C16G—C18G—H18E	108.8
C12E—C13E—H13A	119.4	N2G—C18G—H18F	108.8
C14E—C13E—H13A	119.4	C16G—C18G—H18F	108.8
C13E—C14E—C9E	118.7 (3)	H18E—C18G—H18F	107.7
C13E—C14E—N1E	119.8 (2)	N2G—C19G—H19G	109.5
C9E—C14E—N1E	121.4 (2)	N2G—C19G—H19H	109.5
N1E—C15E—C16E	111.4 (2)	H19G—C19G—H19H	109.5
N1E—C15E—H15A	109.4	N2G—C19G—H19I	109.5
C16E—C15E—H15A	109.4	H19G—C19G—H19I	109.5
N1E—C15E—H15B	109.4	H19H—C19G—H19I	109.5
C16E—C15E—H15B	109.4	N2G—C20G—H20G	109.5
H15A—C15E—H15B	108.0	N2G—C20G—H20H	109.5
C15E—C16E—C18E	109.2 (2)	H20G—C20G—H20H	109.5
C15E—C16E—C17E	109.8 (2)	N2G—C20G—H20I	109.5
C18E—C16E—C17E	110.6 (2)	H20G—C20G—H20I	109.5
C15E—C16E—H16A	109.1	H20H—C20G—H20I	109.5
C18E—C16E—H16A	109.1	C1H—N1H—C14H	120.3 (2)
C17E—C16E—H16A	109.1	C1H—N1H—C15H	115.8 (2)
C16E—C17E—H17A	109.5	C14H—N1H—C15H	118.7 (2)
C16E—C17E—H17B	109.5	C20H—N2H—C19H	110.8 (2)
H17A—C17E—H17B	109.5	C20H—N2H—C18H	109.3 (2)
C16E—C17E—H17C	109.5	C19H—N2H—C18H	112.5 (2)
H17A—C17E—H17C	109.5	C20H—N2H—H2HB	108.0
H17B—C17E—H17C	109.5	C19H—N2H—H2HB	108.0
N2E—C18E—C16E	115.77 (19)	C18H—N2H—H2HB	108.0
N2E—C18E—H18A	108.3	C2H—C1H—C6H	119.1 (2)
C16E—C18E—H18A	108.3	C2H—C1H—N1H	121.2 (2)
N2E—C18E—H18B	108.3	C6H—C1H—N1H	119.6 (2)
C16E—C18E—H18B	108.3	C3H—C2H—C1H	121.2 (2)
H18A—C18E—H18B	107.4	C3H—C2H—H2HA	119.4
N2E—C19E—H19A	109.5	C1H—C2H—H2HA	119.4
N2E—C19E—H19B	109.5	C2H—C3H—C4H	119.6 (2)
H19A—C19E—H19B	109.5	C2H—C3H—H3HA	120.2
N2E—C19E—H19C	109.5	C4H—C3H—H3HA	120.2
H19A—C19E—H19C	109.5	C5H—C4H—C3H	119.4 (2)
H19B—C19E—H19C	109.5	C5H—C4H—H4HA	120.3
N2E—C20E—H20A	109.5	C3H—C4H—H4HA	120.3
N2E—C20E—H20B	109.5	C4H—C5H—C6H	121.8 (2)
H20A—C20E—H20B	109.5	C4H—C5H—H5HA	119.1
N2E—C20E—H20C	109.5	C6H—C5H—H5HA	119.1
H20A—C20E—H20C	109.5	C5H—C6H—C1H	118.7 (2)
H20B—C20E—H20C	109.5	C5H—C6H—C7H	123.6 (2)
C14F—N1F—C1F	117.79 (19)	C1H—C6H—C7H	117.6 (2)
C14F—N1F—C15F	116.4 (2)	C6H—C7H—C8H	112.3 (2)
C1F—N1F—C15F	117.94 (19)	C6H—C7H—H7HA	109.1
C20F—N2F—C19F	110.8 (2)	C8H—C7H—H7HA	109.1
C20F—N2F—C18F	111.8 (2)	C6H—C7H—H7HB	109.1
C19F—N2F—C18F	109.14 (18)	C8H—C7H—H7HB	109.1
C20F—N2F—H2FB	108.3	H7HA—C7H—H7HB	107.9
C19F—N2F—H2FB	108.3	C7H—C8H—C9H	118.4 (2)
C18F—N2F—H2FB	108.3	C7H—C8H—H8HA	107.7
C2F—C1F—C6F	119.0 (2)	C9H—C8H—H8HA	107.7
C2F—C1F—N1F	119.6 (2)	C7H—C8H—H8HB	107.7
C6F—C1F—N1F	121.3 (2)	C9H—C8H—H8HB	107.7
C3F—C2F—C1F	121.5 (2)	H8HA—C8H—H8HB	107.1
C3F—C2F—H2FA	119.3	C10H—C9H—C14H	117.4 (3)
C1F—C2F—H2FA	119.3	C10H—C9H—C8H	116.2 (3)
C4F—C3F—C2F	119.7 (3)	C14H—C9H—C8H	126.4 (2)
C4F—C3F—H3FA	120.2	C11H—C10H—C9H	123.2 (3)
C2F—C3F—H3FA	120.2	C11H—C10H—H10D	118.4
C5F—C4F—C3F	118.8 (3)	C9H—C10H—H10D	118.4
C5F—C4F—H4FA	120.6	C12H—C11H—C10H	118.7 (3)
C3F—C4F—H4FA	120.6	C12H—C11H—H11D	120.7
C4F—C5F—C6F	123.4 (3)	C10H—C11H—H11D	120.7
C4F—C5F—H5FA	118.3	C11H—C12H—C13H	120.0 (3)
C6F—C5F—H5FA	118.3	C11H—C12H—H12D	120.0
C5F—C6F—C1F	117.5 (2)	C13H—C12H—H12D	120.0
C5F—C6F—C7F	116.2 (2)	C12H—C13H—C14H	121.8 (3)
C1F—C6F—C7F	126.2 (2)	C12H—C13H—H13D	119.1
C8F—C7F—C6F	118.5 (2)	C14H—C13H—H13D	119.1
C8F—C7F—H7FA	107.7	C13H—C14H—C9H	118.8 (2)
C6F—C7F—H7FA	107.7	C13H—C14H—N1H	119.6 (3)
C8F—C7F—H7FB	107.7	C9H—C14H—N1H	121.5 (2)
C6F—C7F—H7FB	107.7	N1H—C15H—C16H	111.01 (18)
H7FA—C7F—H7FB	107.1	N1H—C15H—H15G	109.4
C9F—C8F—C7F	111.6 (2)	C16H—C15H—H15G	109.4
C9F—C8F—H8FA	109.3	N1H—C15H—H15H	109.4
C7F—C8F—H8FA	109.3	C16H—C15H—H15H	109.4
C9F—C8F—H8FB	109.3	H15G—C15H—H15H	108.0
C7F—C8F—H8FB	109.3	C18H—C16H—C17H	112.0 (2)
H8FA—C8F—H8FB	108.0	C18H—C16H—C15H	107.05 (18)
C14F—C9F—C10F	118.5 (3)	C17H—C16H—C15H	110.61 (19)
C14F—C9F—C8F	118.9 (2)	C18H—C16H—H16D	109.0
C10F—C9F—C8F	122.6 (3)	C17H—C16H—H16D	109.0
C11F—C10F—C9F	121.5 (3)	C15H—C16H—H16D	109.0
C11F—C10F—H10B	119.3	C16H—C17H—H17J	109.5
C9F—C10F—H10B	119.3	C16H—C17H—H17K	109.5
C12F—C11F—C10F	119.5 (3)	H17J—C17H—H17K	109.5
C12F—C11F—H11B	120.3	C16H—C17H—H17L	109.5
C10F—C11F—H11B	120.3	H17J—C17H—H17L	109.5
C13F—C12F—C11F	119.7 (3)	H17K—C17H—H17L	109.5
C13F—C12F—H12B	120.2	N2H—C18H—C16H	114.64 (18)
C11F—C12F—H12B	120.2	N2H—C18H—H18G	108.6
C12F—C13F—C14F	121.3 (3)	C16H—C18H—H18G	108.6
C12F—C13F—H13B	119.3	N2H—C18H—H18H	108.6
C14F—C13F—H13B	119.3	C16H—C18H—H18H	108.6
C13F—C14F—C9F	119.5 (3)	H18G—C18H—H18H	107.6
C13F—C14F—N1F	122.6 (2)	N2H—C19H—H19J	109.5
C9F—C14F—N1F	117.9 (2)	N2H—C19H—H19K	109.5
N1F—C15F—C16F	110.8 (2)	H19J—C19H—H19K	109.5
N1F—C15F—H15C	109.5	N2H—C19H—H19L	109.5
C16F—C15F—H15C	109.5	H19J—C19H—H19L	109.5
N1F—C15F—H15D	109.5	H19K—C19H—H19L	109.5
C16F—C15F—H15D	109.5	N2H—C20H—H20J	109.5
H15C—C15F—H15D	108.1	N2H—C20H—H20K	109.5
C18F—C16F—C17F	112.4 (2)	H20J—C20H—H20K	109.5
C18F—C16F—C15F	108.10 (19)	N2H—C20H—H20L	109.5
C17F—C16F—C15F	109.1 (2)	H20J—C20H—H20L	109.5
C18F—C16F—H16B	109.1	H20K—C20H—H20L	109.5
			
O2A—C1A—C2A—C3A	−163.1 (3)	C1F—N1F—C14F—C9F	71.6 (3)
O1A—C1A—C2A—C3A	16.6 (4)	C15F—N1F—C14F—C9F	−139.7 (2)
C1A—C2A—C3A—C4A	0.3 (4)	C14F—N1F—C15F—C16F	67.3 (3)
C2A—C3A—C4A—O4A	162.5 (3)	C1F—N1F—C15F—C16F	−144.1 (2)
C2A—C3A—C4A—O3A	−18.1 (4)	N1F—C15F—C16F—C18F	71.8 (3)
O2B—C1B—C2B—C3B	−162.4 (3)	N1F—C15F—C16F—C17F	−165.6 (2)
O1B—C1B—C2B—C3B	17.1 (4)	C17F—C16F—C18F—N2F	73.5 (3)
C1B—C2B—C3B—C4B	−0.8 (5)	C15F—C16F—C18F—N2F	−166.03 (19)
C2B—C3B—C4B—O4B	163.4 (3)	C20F—N2F—C18F—C16F	76.0 (3)
C2B—C3B—C4B—O3B	−16.5 (4)	C19F—N2F—C18F—C16F	−161.0 (2)
O2C—C1C—C2C—C3C	−173.9 (3)	C14G—N1G—C1G—C6G	−72.0 (3)
O1C—C1C—C2C—C3C	5.0 (4)	C15G—N1G—C1G—C6G	132.2 (2)
C1C—C2C—C3C—C4C	−1.0 (5)	C14G—N1G—C1G—C2G	110.8 (3)
C2C—C3C—C4C—O4C	177.9 (3)	C15G—N1G—C1G—C2G	−45.0 (3)
C2C—C3C—C4C—O3C	−2.6 (4)	C6G—C1G—C2G—C3G	−2.5 (4)
O2D—C1D—C2D—C3D	177.3 (3)	N1G—C1G—C2G—C3G	174.6 (2)
O1D—C1D—C2D—C3D	−2.7 (4)	C1G—C2G—C3G—C4G	2.7 (4)
C1D—C2D—C3D—C4D	−2.0 (5)	C2G—C3G—C4G—C5G	−1.4 (4)
C2D—C3D—C4D—O4D	−172.6 (3)	C3G—C4G—C5G—C6G	−0.1 (4)
C2D—C3D—C4D—O3D	6.8 (4)	C2G—C1G—C6G—C5G	1.0 (4)
C14E—N1E—C1E—C2E	−107.9 (3)	N1G—C1G—C6G—C5G	−176.2 (2)
C15E—N1E—C1E—C2E	40.9 (3)	C2G—C1G—C6G—C7G	−177.2 (2)
C14E—N1E—C1E—C6E	72.4 (3)	N1G—C1G—C6G—C7G	5.6 (3)
C15E—N1E—C1E—C6E	−138.8 (2)	C4G—C5G—C6G—C1G	0.2 (4)
C6E—C1E—C2E—C3E	0.7 (4)	C4G—C5G—C6G—C7G	178.4 (3)
N1E—C1E—C2E—C3E	−179.0 (2)	C1G—C6G—C7G—C8G	67.8 (3)
C1E—C2E—C3E—C4E	−1.3 (4)	C5G—C6G—C7G—C8G	−110.3 (3)
C2E—C3E—C4E—C5E	1.0 (4)	C6G—C7G—C8G—C9G	−65.4 (3)
C3E—C4E—C5E—C6E	0.0 (4)	C7G—C8G—C9G—C10G	−166.5 (3)
C4E—C5E—C6E—C1E	−0.6 (4)	C7G—C8G—C9G—C14G	10.3 (4)
C4E—C5E—C6E—C7E	177.6 (3)	C14G—C9G—C10G—C11G	0.4 (4)
C2E—C1E—C6E—C5E	0.3 (4)	C8G—C9G—C10G—C11G	177.5 (3)
N1E—C1E—C6E—C5E	180.0 (2)	C9G—C10G—C11G—C12G	−1.0 (4)
C2E—C1E—C6E—C7E	−178.0 (2)	C10G—C11G—C12G—C13G	0.5 (4)
N1E—C1E—C6E—C7E	1.7 (4)	C11G—C12G—C13G—C14G	0.5 (4)
C5E—C6E—C7E—C8E	111.5 (3)	C12G—C13G—C14G—C9G	−1.1 (4)
C1E—C6E—C7E—C8E	−70.3 (3)	C12G—C13G—C14G—N1G	−179.8 (2)
C6E—C7E—C8E—C9E	57.2 (4)	C10G—C9G—C14G—C13G	0.7 (4)
C7E—C8E—C9E—C10E	174.6 (2)	C8G—C9G—C14G—C13G	−176.1 (3)
C7E—C8E—C9E—C14E	−5.0 (4)	C10G—C9G—C14G—N1G	179.3 (2)
C14E—C9E—C10E—C11E	−0.8 (4)	C8G—C9G—C14G—N1G	2.5 (4)
C8E—C9E—C10E—C11E	179.5 (3)	C1G—N1G—C14G—C13G	−132.7 (3)
C9E—C10E—C11E—C12E	−1.0 (4)	C15G—N1G—C14G—C13G	22.6 (4)
C10E—C11E—C12E—C13E	0.9 (5)	C1G—N1G—C14G—C9G	48.7 (4)
C11E—C12E—C13E—C14E	1.0 (4)	C15G—N1G—C14G—C9G	−156.0 (2)
C12E—C13E—C14E—C9E	−2.8 (4)	C14G—N1G—C15G—C16G	70.4 (3)
C12E—C13E—C14E—N1E	174.9 (2)	C1G—N1G—C15G—C16G	−133.5 (2)
C10E—C9E—C14E—C13E	2.6 (3)	N1G—C15G—C16G—C18G	−163.6 (2)
C8E—C9E—C14E—C13E	−177.8 (2)	N1G—C15G—C16G—C17G	73.5 (3)
C10E—C9E—C14E—N1E	−174.9 (2)	C20G—N2G—C18G—C16G	−173.2 (2)
C8E—C9E—C14E—N1E	4.6 (4)	C19G—N2G—C18G—C16G	−50.1 (3)
C1E—N1E—C14E—C13E	121.5 (3)	C17G—C16G—C18G—N2G	−60.5 (3)
C15E—N1E—C14E—C13E	−26.5 (3)	C15G—C16G—C18G—N2G	176.9 (2)
C1E—N1E—C14E—C9E	−60.9 (3)	C14H—N1H—C1H—C2H	111.1 (3)
C15E—N1E—C14E—C9E	151.0 (2)	C15H—N1H—C1H—C2H	−43.2 (3)
C14E—N1E—C15E—C16E	−146.8 (2)	C14H—N1H—C1H—C6H	−73.4 (3)
C1E—N1E—C15E—C16E	64.9 (3)	C15H—N1H—C1H—C6H	132.3 (2)
N1E—C15E—C16E—C18E	69.9 (3)	C6H—C1H—C2H—C3H	−3.4 (3)
N1E—C15E—C16E—C17E	−168.7 (2)	N1H—C1H—C2H—C3H	172.1 (2)
C20E—N2E—C18E—C16E	−160.2 (2)	C1H—C2H—C3H—C4H	2.0 (3)
C19E—N2E—C18E—C16E	76.4 (3)	C2H—C3H—C4H—C5H	0.8 (3)
C15E—C16E—C18E—N2E	−165.9 (2)	C3H—C4H—C5H—C6H	−2.2 (4)
C17E—C16E—C18E—N2E	73.2 (3)	C4H—C5H—C6H—C1H	0.7 (4)
C14F—N1F—C1F—C2F	123.3 (2)	C4H—C5H—C6H—C7H	178.4 (2)
C15F—N1F—C1F—C2F	−24.8 (3)	C2H—C1H—C6H—C5H	2.1 (4)
C14F—N1F—C1F—C6F	−59.2 (3)	N1H—C1H—C6H—C5H	−173.6 (2)
C15F—N1F—C1F—C6F	152.6 (2)	C2H—C1H—C6H—C7H	−175.8 (2)
C6F—C1F—C2F—C3F	−3.5 (4)	N1H—C1H—C6H—C7H	8.6 (3)
N1F—C1F—C2F—C3F	173.9 (2)	C5H—C6H—C7H—C8H	−112.9 (3)
C1F—C2F—C3F—C4F	0.2 (4)	C1H—C6H—C7H—C8H	64.8 (3)
C2F—C3F—C4F—C5F	2.1 (4)	C6H—C7H—C8H—C9H	−65.2 (3)
C3F—C4F—C5F—C6F	−1.1 (4)	C7H—C8H—C9H—C10H	−166.9 (3)
C4F—C5F—C6F—C1F	−2.1 (4)	C7H—C8H—C9H—C14H	11.0 (4)
C4F—C5F—C6F—C7F	177.2 (2)	C14H—C9H—C10H—C11H	−1.1 (4)
C2F—C1F—C6F—C5F	4.4 (3)	C8H—C9H—C10H—C11H	177.0 (3)
N1F—C1F—C6F—C5F	−173.1 (2)	C9H—C10H—C11H—C12H	−0.2 (5)
C2F—C1F—C6F—C7F	−174.9 (2)	C10H—C11H—C12H—C13H	0.8 (4)
N1F—C1F—C6F—C7F	7.7 (4)	C11H—C12H—C13H—C14H	−0.1 (4)
C5F—C6F—C7F—C8F	168.2 (2)	C12H—C13H—C14H—C9H	−1.2 (4)
C1F—C6F—C7F—C8F	−12.5 (4)	C12H—C13H—C14H—N1H	−179.7 (3)
C6F—C7F—C8F—C9F	62.7 (3)	C10H—C9H—C14H—C13H	1.7 (4)
C7F—C8F—C9F—C14F	−70.6 (3)	C8H—C9H—C14H—C13H	−176.1 (3)
C7F—C8F—C9F—C10F	108.7 (3)	C10H—C9H—C14H—N1H	−179.8 (2)
C14F—C9F—C10F—C11F	−1.8 (4)	C8H—C9H—C14H—N1H	2.4 (4)
C8F—C9F—C10F—C11F	178.9 (2)	C1H—N1H—C14H—C13H	−133.3 (3)
C9F—C10F—C11F—C12F	0.0 (4)	C15H—N1H—C14H—C13H	20.3 (3)
C10F—C11F—C12F—C13F	1.3 (4)	C1H—N1H—C14H—C9H	48.2 (3)
C11F—C12F—C13F—C14F	−0.9 (4)	C15H—N1H—C14H—C9H	−158.2 (2)
C12F—C13F—C14F—C9F	−0.9 (4)	C1H—N1H—C15H—C16H	−134.1 (2)
C12F—C13F—C14F—N1F	−179.6 (2)	C14H—N1H—C15H—C16H	71.2 (3)
C10F—C9F—C14F—C13F	2.2 (4)	N1H—C15H—C16H—C18H	−165.0 (2)
C8F—C9F—C14F—C13F	−178.4 (2)	N1H—C15H—C16H—C17H	72.6 (2)
C10F—C9F—C14F—N1F	−179.0 (2)	C20H—N2H—C18H—C16H	−172.8 (2)
C8F—C9F—C14F—N1F	0.3 (4)	C19H—N2H—C18H—C16H	−49.3 (3)
C1F—N1F—C14F—C13F	−109.6 (3)	C17H—C16H—C18H—N2H	−59.8 (3)
C15F—N1F—C14F—C13F	39.0 (3)	C15H—C16H—C18H—N2H	178.7 (2)

### Hydrogen-bond geometry (Å, °)

**Table:**

*D*—H···*A*	*D*—H	H···*A*	*D*···*A*	*D*—H···*A*
O3A—H3A···O1A	0.84	1.63	2.437 (2)	161
O3B—H3B···O1B	0.84	1.61	2.437 (2)	167
O3C—H3C···O1C	0.84	1.58	2.417 (3)	177
O3D—H3D···O1D	0.84	1.58	2.422 (3)	178
N2E—H2EB···O2A	0.93	1.86	2.736 (3)	156
N2F—H2FB···O2B	0.93	1.86	2.737 (3)	156
N2G—H2GB···O2C	0.93	1.87	2.760 (3)	158
N2H—H2HB···O4D	0.93	1.87	2.751 (3)	158
N2H—H2HB···O3D	0.93	2.63	3.312 (3)	131
C17E—H17C···O2A	0.98	2.63	3.560 (4)	159
C18E—H18B···O2D^i^	0.99	2.28	3.271 (3)	174
C18F—H18C···O4C^ii^	0.99	2.31	3.293 (3)	174
C18G—H18E···O2B	0.99	2.42	3.404 (3)	171
C18G—H18F···O3C^iii^	0.99	2.54	3.453 (3)	154
C18G—H18F···O4C^iii^	0.99	2.53	3.432 (3)	152
C18H—H18G···O2A	0.99	2.42	3.402 (3)	173
C18H—H18H···O1D^iv^	0.99	2.55	3.473 (3)	155
C18H—H18H···O2D^iv^	0.99	2.53	3.435 (3)	152
C19E—H19A···O4D	0.98	2.62	3.198 (3)	118
C19F—H19E···O4B^iv^	0.98	2.54	3.464 (3)	158
C19G—H19I···O4B^v^	0.98	2.53	3.476 (3)	163
C19H—H19K···O4A^ii^	0.98	2.56	3.516 (3)	164
C20E—H20A···O4A^vi^	0.98	2.56	3.488 (3)	159
C20G—H20H···O4C^iii^	0.98	2.60	3.479 (3)	149
C20H—H20K···O2D^iv^	0.98	2.63	3.497 (3)	148

Symmetry codes: (i) *x*+1/2, −*y*+3/2, *z*; (ii) *x*−1/2, −*y*+1/2, *z*; (iii) *x*−1/2, −*y*−1/2, *z*; (iv) *x*+1/2, −*y*+1/2, *z*; (v) *x*+1/2, −*y*−1/2, *z*; (vi) *x*−1/2, −*y*+3/2, *z*.
